# Hyaluronidase 1 deficiency decreases bone mineral density in mice

**DOI:** 10.1038/s41598-022-14473-7

**Published:** 2022-06-16

**Authors:** Emeline Puissant, Florentine Gilis, Virginie Tevel, Jean-Michel Vandeweerd, Bruno Flamion, Michel Jadot, Marielle Boonen

**Affiliations:** 1 URPhyM, Physiological Chemistry laboratory, NARILIS, University of Namur, Namur, Belgium; 2 URPhyM, Physiology and Pharmacology laboratory, NARILIS, University of Namur, Namur, Belgium; 3 URVI, Integrated Veterinary Research Unit, NARILIS, University of Namur, Namur, Belgium; 4 URPhyM, Intracellular Trafficking Biology, NARILIS, University of Namur, Rue de Bruxelles 61, 5000 Namur, Belgium

**Keywords:** Bone, Glycobiology

## Abstract

Mucopolysaccharidosis IX is a lysosomal storage disorder caused by a deficiency in HYAL1, an enzyme that degrades hyaluronic acid at acidic pH. This disease causes juvenile arthritis in humans and osteoarthritis in the *Hyal1* knockout mouse model. Our past research revealed that HYAL1 is strikingly upregulated (~ 25x) upon differentiation of bone marrow monocytes into osteoclasts. To investigate whether HYAL1 is involved in the differentiation and/or resorption activity of osteoclasts, and in bone remodeling in general, we analyzed several bone parameters in *Hyal1* −/− mice and studied the differentiation and activity of their osteoclasts and osteoblasts when differentiated in vitro. These experiments revealed that, upon aging, HYAL1 deficient mice exhibit reduced femur length and a ~ 15% decrease in bone mineral density compared to wild-type mice. We found elevated osteoclast numbers in the femurs of these mice as well as an increase of the bone resorbing activity of *Hyal1* −/− osteoclasts. Moreover, we detected decreased mineralization by *Hyal1* −/− osteoblasts. Taken together with the observed accumulation of hyaluronic acid in *Hyal1* −/− bones, these results support the premise that the catabolism of hyaluronic acid by osteoclasts and osteoblasts is an intrinsic part of bone remodeling.

## Introduction

Hyaluronic acid (HA), aka hyaluronan, is a high molecular mass (MM) and viscoelastic glycosaminoglycan abundant in soft connective tissues^1^. By comparison, dense connective tissues such as bone only contain low levels of HA^2^. Intriguingly though, it has been reported that incubation of osteoblasts or osteoclasts in the presence of HA (or HA fragments of different MM) modifies their differentiation rate and/or activity in vitro (reviewed in^3^). These studies support that, while not a major structural component of the bone matrix, HA may be an important regulator of bone remodeling. It remains difficult, however, to predict if and how endogenous bone HA participates to bone remodeling in vivo.

We have recently found that the lysosomal hyaluronidase HYAL1 is highly upregulated upon differentiation of macrophages into osteoclasts^4^. These giant multinucleated cells polarize when they come in contact with bone matrix and form an extracellular lacuna by sealing their apical membrane to bone components. The bone matrix is then digested within this lacuna, notably through the exocytosis of acid hydrolases contained in secretory lysosomes and concomitant acidification of the lacuna by the lysosomal vATPase^5,6^. Interestingly, aside from HYAL1, only two other lysosomal enzymes are highly upregulated upon osteoclastogenesis, i.e. cathepsin K and TRAP (Tartrate-Resistant Acid Phosphatase), which are both central actors of bone remodeling^7^. Based on this observation and on the previous reports that HA affects bone cell behavior^3^, we postulated that HYAL1 could be another player in this process, possibly through the regulation of HA concentration and/or molecular mass in the bone environment. Of note, *Hyal1* knockout mice exhibit increased HA levels particularly in their liver, lymph nodes, and serum but without change in HA MM pattern^8^, suggesting that HYAL1 is not a major contributor to the distribution of high- vs low-MM HA within the organism. *Hyal1* knockout mice also suffer from osteoarthritis^9^, which is in line with the skeletal alterations seen in humans suffering from mucopolysaccharidosis IX, a HYAL1 deficiency of genetic origin^9,10^.Some patients with congenital HYAL1 deficiency exhibit juvenile idiopathic arthritis with proliferative synovitis, macrophage infiltration of the synovial membrane, synovial effusion and articular pain^11,12^. Osteoporosis has not been reported in the few patients with mucopolysaccharidosis IX up to now, though it is worth noting that the published clinical assessments were conducted on young patients (11–21 years old).

To investigate the putative role of HYAL1 in bone remodeling, we analyzed the in vitro differentiation and bone resorbing activity of osteoclasts derived from *Hyal1* −/− mouse bone marrow monocytes, tested *Hyal1 -/-* osteoblast activity when cultured in vitro, and investigated several bone parameters of aged (12- and 18-month-old) *Hyal1* −/− mice. Our results reveal that HYAL1 deficiency leads to a decrease of bone mineral density in mouse femurs, associated with decreased femur length, increased osteoclast numbers and resorption activity, and reduced osteoblast activity.

## Results

### *Hyal1* −/− osteoclasts differentiated in vitro exhibit increased resorption activity

**Figure 1:**
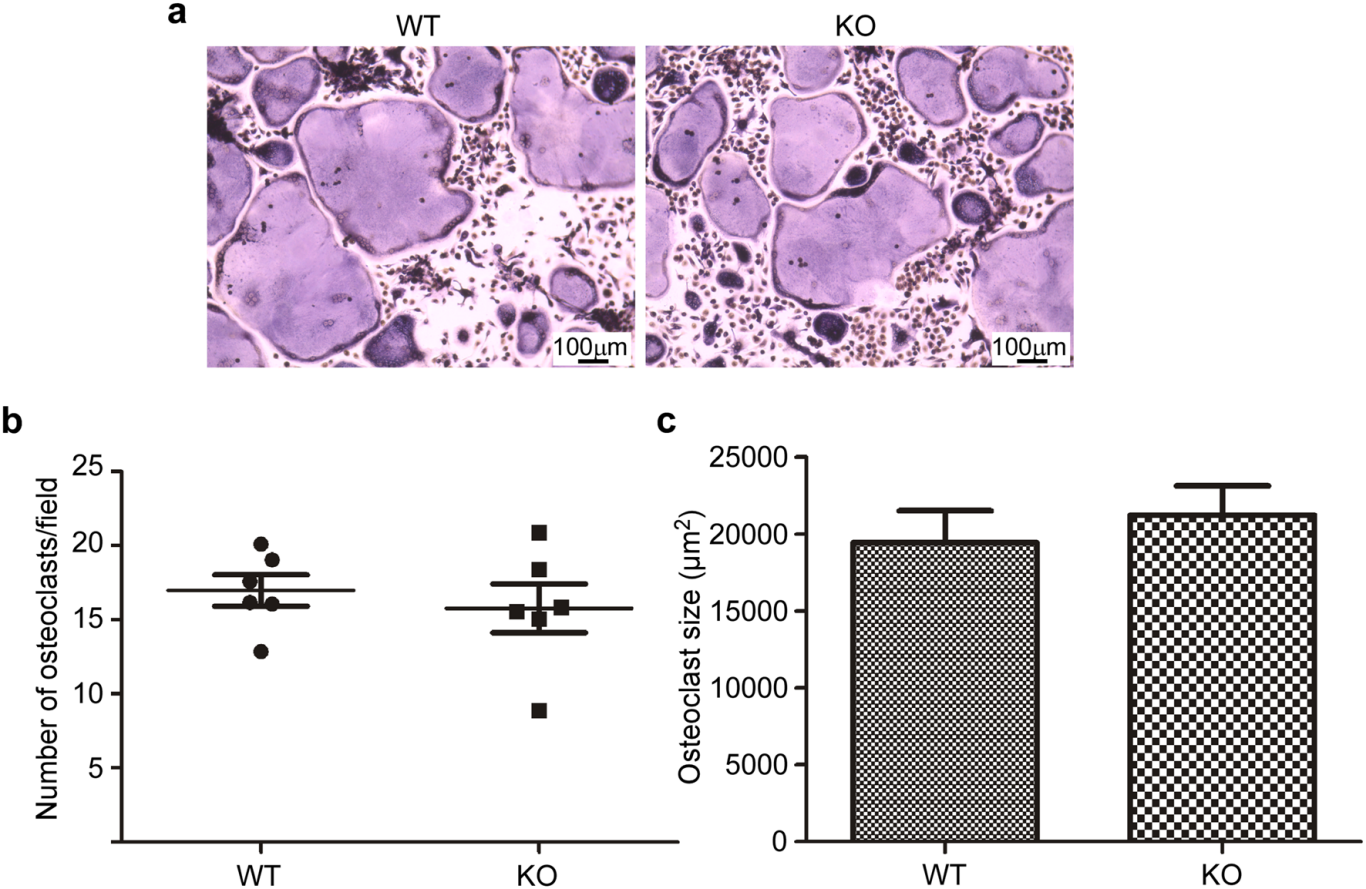
*Hyal1* inactivation does not affect the differentiation of osteoclasts in vitro. (**a**) WT and *Hyal1* knockout (KO) osteoclasts were differentiated for 7 days on glass coverslips before staining of the osteoclast marker TRAP (Fast Garnet GBC method). (**b**) The osteoclasts were counted as the number of TRAP-positive cells containing 3 or more nuclei. The quantifications were made on 25 images collected from 2 technical replicates for each mouse (n = 6 mice in each group). The graph shows the number of osteoclasts per field for both genotypes (mean ± SEM). (**c**) The size (surface) of WT and KO osteoclasts differentiated on glass coverslip was measured as the average of 60 cells for each mouse (n = 5 mice per group). The graph shows the means ± SEM. Two-tailed Mann–Whitney U tests indicate that there is no difference between the groups for these measurements (**b**,**c**).

We isolated monocytes from the bone marrow of 7-month-old wild-type (WT) and *Hyal1* −/− mice^4,9^ and cultured them in vitro for 5 days in the presence of M-CSF (Macrophage-Colony Stimulating Factor) to stimulate their proliferation, survival and differentiation into macrophages^13^. Then, RANKL (Receptor Activator of Nuclear factor Kappa-B Ligand) was added to induce osteoclastogenesis^14^. After 7 days of culture on a flat glass surface, the number and size of TRAP-positive multinucleated cells (with 3 or more nuclei) were assessed. No difference was found between WT and *Hyal1* −/− groups (Fig. 1a–c), indicating that the knockout of *Hyal1* does not slow down nor accelerate the differentiation process of osteoclasts in vitro.

**Figure 2:**
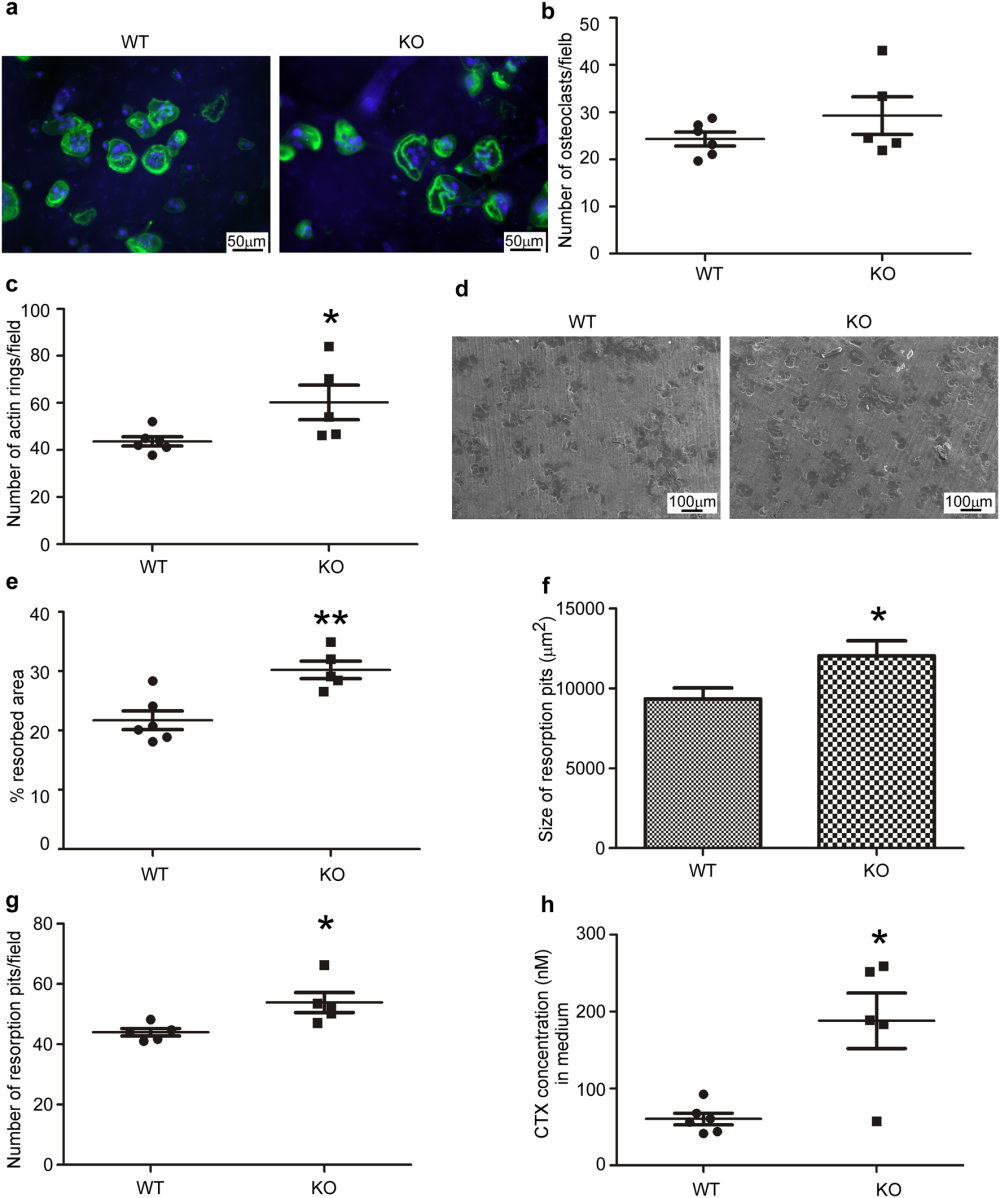
Inactivation of HYAL1 increases the resorption activity of osteoclasts. (**a**) After 10 days of differentiation on bovine bone slices, WT or *Hyal1* −/− osteoclasts were fixed with paraformaldehyde. Actin rings and nuclei were then stained with Alexa 488-phalloidin (green) and Hoechst 33258 (blue), respectively. (**b**) The number of polarized osteoclasts was quantified on 2 bone slices for each mouse (with n ≥ 5 mice in each group and min 5 images per bone slices). The graph shows mean numbers of osteoclasts per field ± SEM. (**c**) The same images were used to compare the number of actin rings between WT and *Hyal1* −/− conditions. Results are expressed as mean numbers per field ± SEM. (**d**) After removal of the osteoclasts, the resorption pits were observed by scanning electron microscopy. (**e**,**f**) Quantifications of the percentage of the resorbed area (**e**) and of the number of resorption pits per field (**f**) were conducted based on 10 images per bone slice (with 2 bone slices for each mouse and n = 5 mice per genotype). (**g**) The average size of resorption pits was assessed from the measurement of at least 60 pits for each mouse (n = 5 mice per group). (**h**) CTX released from the bovine bone slices upon resorption by osteoclasts were measured in the culture supernatants using an ELISA assay. To do so, conditioned media were collected between day 8 and day 10 after plating and differentiation of the macrophages on bone slices (n ≥ 5 mice per group, and technical replicates were quantified). The graphs show the means ± SEM. *p < 0.05, **p < 0.01 (two-tailed Mann–Whitney U tests).

Next, bone marrow-derived monocytes were differentiated into osteoclasts on thin bone slices obtained from bovine nasal bone to assess their polarization and bone matrix resorption activity, as described in^15^. Staining of the nuclei with Hoechst 33,258 and incubation with Alexa 488-phalloidin (to label the actin rings that delimit resorption lacunae) pointed out that the number of differentiated and polarized osteoclasts is similar, irrespective of the WT or *Hyal1* −/− background (Fig. 2a,b). However, the number of actin rings was found increased (Fig. 2c, p < 0.05). Moreover, scanning electron microscopy analysis of resorbed bone area, as well an assay of the C-terminal Telopeptide of type I-collagen degradation products (CTX, i.e. bone degradation products) released in the culture medium, revealed that the bone resorbing activity of *Hyal1* −/− osteoclasts is increased relative to WT osteoclasts (Fig. 2d–f). Indeed, the percentage of resorbed area represented 22 ± 4% for WT cells, compared to 30 ± 3% for KO osteoclasts (Fig. 2d,e, p < 0.01). Concomitantly, the latter released more CTX fragments than the former (2.5-fold increase, Fig. 2h, p < 0.05). In addition, the number and size of resorption pits were found elevated for *Hyal1* −/− osteoclasts (Fig. 2f,g, p < 0.05).

### HYAL1 deficiency causes HA accumulation in mouse femurs

**Figure 3:**
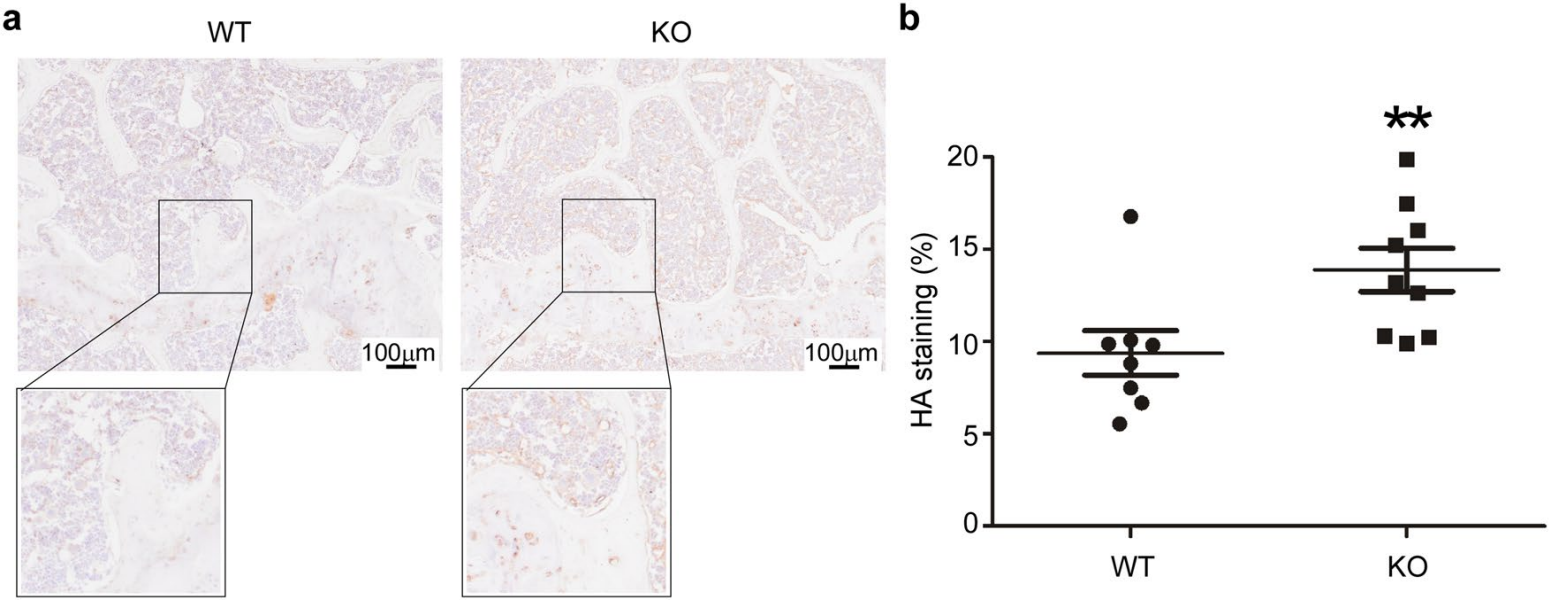
HA accumulates in the femurs of *Hyal1* −/− mice. (**a**) Histological sections of WT and *Hyal1* −/−femurs were stained for HA using a biotinylated HABP followed by incubation with streptavidin HRP and DAB (3,3ʹ-diaminobenzidine) oxidation. Tissue sections were counterstained with hemalun. Inlets show zoomed areas (**b**) The extent of the HA staining was quantified and expressed as a percentage of the total area (in n ≥ 8 mice for each group). The graph shows the means ± SEM. **p < 0.01 (two-tailed Mann–Whitney U test).

To investigate whether the inactivation of *Hyal1* alters the level of HA in bone, we stained HA using HABP (HA-binding protein) on femur sections of 18-month-old mice. This analysis showed a significant increase of the HA staining in the *Hyal1* −/− mouse femurs (Fig. 3a,b).

### *Hyal1* −/− mice exhibit decreased femur length and decreased bone mineral density

We wondered whether the increase of bone resorption activity detected for *Hyal1* −/− osteoclasts in vitro translates into an alteration of bone remodeling in vivo. Using peripheral Quantitative Computed Tomography (pQCT), we measured the bone mineral density (BMD) of right femurs isolated from 1-year-old male mice of wild-type (WT) and HYAL1 deficient (*Hyal1* −/−) mice. (Table 1, n ≥ 6 per group). Three different areas of the bones located at 17.5% and 22% (metaphysis), and at 50% (diaphysis) distance from the knee joint space (distal end) were scanned. Two-tailed Mann–Whitney U statistical tests revealed a significant, ~ 25% decrease of trabecular BMD at 17.5 and 22% in the metaphysis of *Hyal1* −/− femurs (p < 0.01). Total BMD was decreased by ~ 15% at these locations. A significant decrease of cortical BMD was also measured in their diaphysis (p = 0.01) but it should be noted that due to voxel size, this measurement is less accurate.

**Table 1:** Bone mineral density (BMD; mg/cm^3^) of right femurs isolated from 1-year-old WT or *Hyal1* −/− male mice. The BMD was measured using peripheral Quantitative Computed Tomography at 17.5, 22, 50% distance from the distal extremity of the femurs. Two-tailed Mann–Whitney U tests were applied to the results. *p < 0.05, **p < 0.01.

Mean (mg/cm^3^) ± SEM	WT (n = 7)	*Hyal1* −/− (n = 6)	p value
**Metaphysis (17.5%)**			
Total BMD	357.2 ± 9.2	305.7 ± 7	0.0047**
Trabecular BMD	250.2 ± 7.7	182.7 ± 14	0.0023**
Cortical BMD	501.0 ± 11.5	478.0 ± 7.8	0.107
**Metaphysis (22%)**			
Total BMD	400.4 ± 8.7	351.7 ± 7	0.0047**
Trabecular BMD	283.0 ± 7.8	215.1 ± 6.7	0.0012**
Cortical BMD	545.1 ± 10.2	510.8 ± 6.3	0.051
**Diaphysis (50%)**			
Cortical BMD	674.3 ± 10.4	626.8 ± 8	0.01*

**Figure 4:**
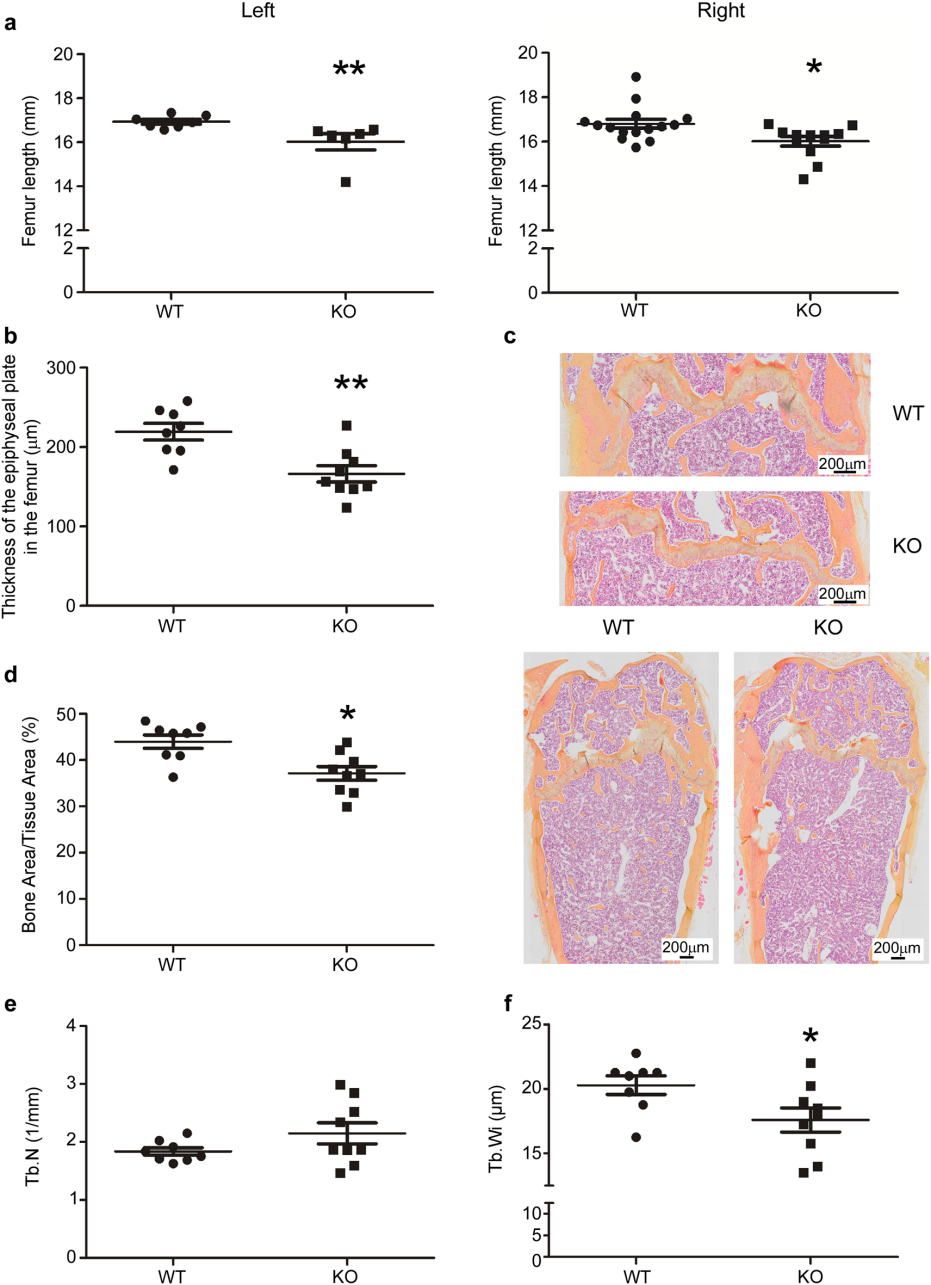
Femur length, thickness of the epiphyseal plate, percentage of bone matrix, and trabecular width in *Hyal1* −/− (KO) mice are decreased compared to WT mice. (**a**) Left (n ≥ 6) and right (n ≥ 12) femurs were isolated from 1-year-old mice. Their length (in mm) was measured with a digital caliper. (**b**,**c**) The thickness of the epiphyseal plate at the distal extremity of femurs from 18-month-old mice was quantified on 7 histological sections from each mouse (representative slides shown in **c**). Eight measures were taken across the epiphyseal plate and averaged for each section (n ≥ 8 mice per group). (**c**,**d**) From the same histological slices, we calculated the percentage of bone area (light orange after hematoxylin eosin saffron coloration) compared to the total tissue area (n ≥ 8 mice in each group, 7 sections for each mouse). (**e**,**f**) Analyses of Trabecular Number (Tb.N) (**e**) and Trabecular Width (Tb.Wi) (**f**) in epiphyses (4 bone slices per mice, n ≥ 8 mice per group). The graphs represent the means ± SEM. *p < 0.05, **p < 0.01 (two-tailed Mann–Whitney U tests).

Moreover, we detected a significant decrease of the length of left femurs in *Hyal1* −/− vs WT mice (with p values < 0.05; Fig. 4a). Taken together, these data point out that bone remodeling is altered in *Hyal1* −/− mice and that this alteration leads to decreased BMD and shortened femurs. In accordance with HYAL1 affecting bone growth, histological analyses revealed a significant decrease of the epiphyseal growth plate thickness (Fig. 4b,c).

In addition, a decrease of the bone area ratio relative to the total tissue area in *Hyal1* −/− femurs was detected in bone sections, consistent with decreased BMD (Fig. 4c,d). Trabecular number (Tb.N) in the epiphysis was not changed, but trabecular width (Tb.Wi) was found decreased (Fig. 4e,f).

### Osteoclast numbers are increased in the epiphysis and metaphysis of *Hyal1* −/− mouse femurs whereas osteoblast numbers are unchanged

**Figure 5:**
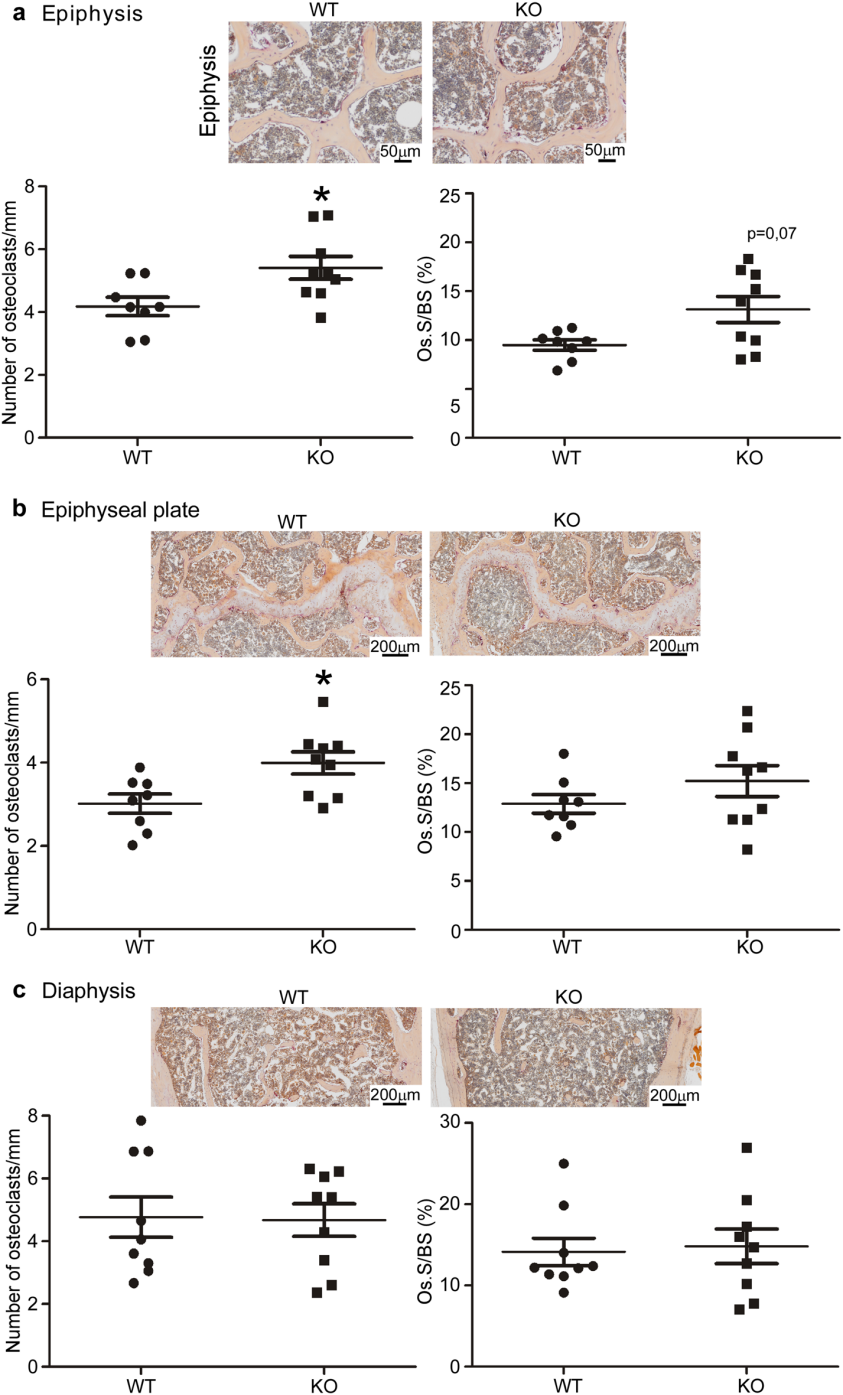
The number of osteoclasts is increased in the epiphysis and metaphysis of *Hyal1* −/− femurs compared to WT femurs. (**a**–**c**) Representative histological sections of distal femurs from 18-month-old male *Hyal1* −/− or WT mice stained for TRAP and counterstained with hemalun. The number of osteoclasts per mm, as well as Osteoclast Surface/Bone Surface (Os.S/BS in %) were calculated on 2 slices for each mouse (with n ≥ 8 for each group). The quantifications were conducted at 3 different positions in the femur: the epiphysis (**a**), the epiphyseal plate (**b**), and the diaphysis (**c**) (approximately at 25–30% distance from the distal end). The graphs represent the means ± SEM. *p < 0.05 (two-tailed Mann–Whitney U tests).

Osteoclasts were identified and counted in femur sections of 18-month-old WT and KO mice through the histochemical detection of TRAP (Fig. 5). This analysis revealed that the number of osteoclasts per mm, and osteoclast surface relative to bone surface (Os.S/BS), are increased in *Hyal1* −/− vs WT mice both at the epiphysis (Fig. 5a**;** p < 0.05 and p = 0.07, respectively). These parameters were also increased at the epiphyseal plate (Fig. 5b) though statistical threshold was only reached for osteoclast number quantifications (p < 0.05). We also analyzed the diaphysis (Fig. 5c) but found no difference in osteoclast numbers or surface at this site.

**Figure 6:**
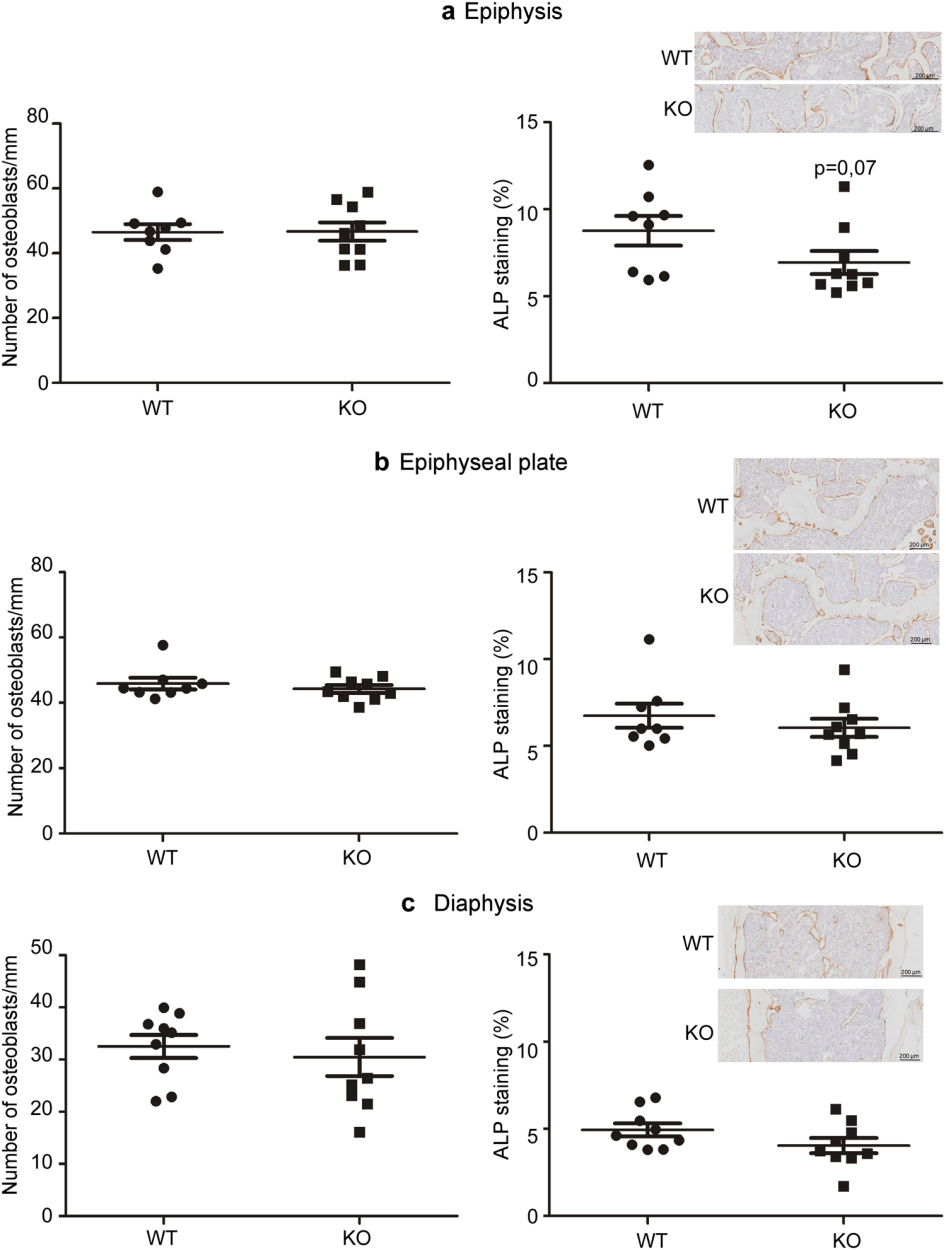
Osteoblast numbers are not modified in HYAL1 deficient femurs, but alkaline phosphatase (ALP) immunostaining is decreased in the epiphysis. (**a**–**c**) The number of osteoblasts per mm and the % of area positive for alkaline phosphatase were quantified on slices at the epiphysis, the epiphyseal plate, and the diaphysis (approximately at a 25–30% distance from the distal end) of 18-month-old mice (n ≥ 8 mice per group). N.B. Osteoblast numbers were counted in HES stained sections (see Fig. 5 for representative micrographies). The graphs show the means ± SEM. *p < 0.05 (two-tailed Mann–Whitney U tests).

Although osteoblasts do not express HYAL1^16^, it has been reported that HA can influence the differentiation and activity of these bone forming cells in vitro^3^. Hence, we also considered that the loss of HYAL1 in osteoclasts could alter osteoblast numbers, possibly through an alteration of the catabolism of HA in *Hyal1* −/− bones. To investigate this possibility, we counted osteoblasts on hematoxylin eosin saffron (HES) stained sections of WT and *Hyal1* −/− femurs, and detected the osteoblast marker alkaline phosphatase by immunohistochemistry (Fig. 6a–c). No difference was detected between mouse groups for osteoblast numbers. However, a decrease of the percentage of stained area was observed in the epiphysis (p = 0.07), suggesting a HYAL1 deficiency may inhibit osteoblast differentiation in vivo.

### Circulating levels of PINP are decreased and CTX levels are increased in 18-month-old *Hyal1* −/− mice

**Figure 7:**
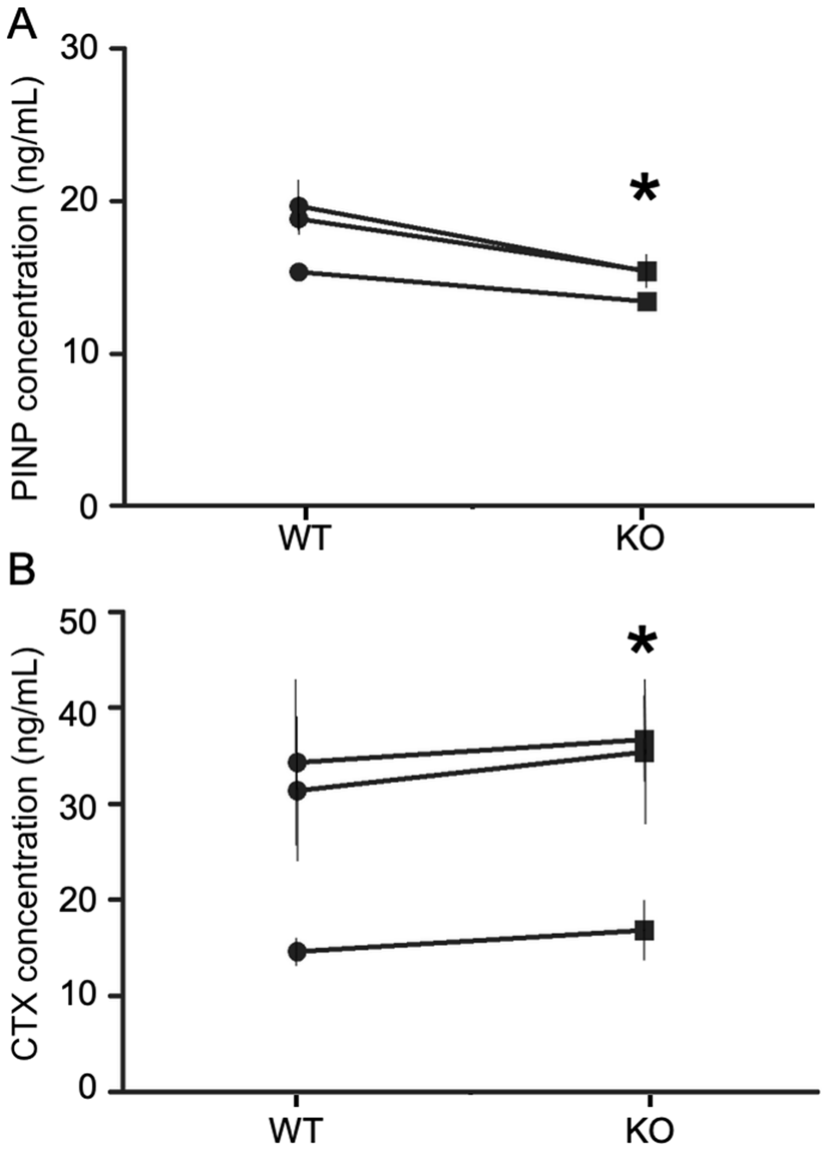
PINP and CTX levels in 18-month-old wild-type and *Hyal1* −/− mice. PINP (Procollagen type I N-terminal propeptide) (**a**) and CTX (C-Terminal Telopeptide of type I-collagen) (**b**) levels were quantified using ELISA assays in 18-month-old mice from both genotypes. Three independent series of mice (with n ≥ 4 for each genotype) were included in three separate experiments. Paired means ± SEM are shown in the graphs for each experiment. *p < 0.05. Paired t-test.

We measured the circulating levels of Procollagen type I N-terminal Propeptide (PINP), a marker of bone formation by osteoblasts, and of the osteoclast resorption marker CTX. Three groups of 18-month-old mice (with n ≥ 4 mice for each genotype in each group) were assessed independently. The results show a statistically significant decrease of PINP levels in *Hyal1* −/− plasma (Fig. 7a). This finding indicates that osteoblast activity is decreased, since osteoblast numbers were found unchanged on *Hyal1* −/− bone sections. In addition, an increase of CTX level was detected in sera of the HYAL1 deficient groups (Fig. 7b). Coupled with the elevated CTX levels detected in the culture medium of *Hyal1* −/− osteoclasts differentiated on bone slices in vitro (cf. Fig. 2h), this result points out that HYAL1 deficiency potentiates the bone resorption activity of osteoclasts*.*

### The knock-down of *Hyal1* impairs the activity of osteoblasts differentiated in vitro from mouse calvariae pre-osteoblasts

**Figure 8:**
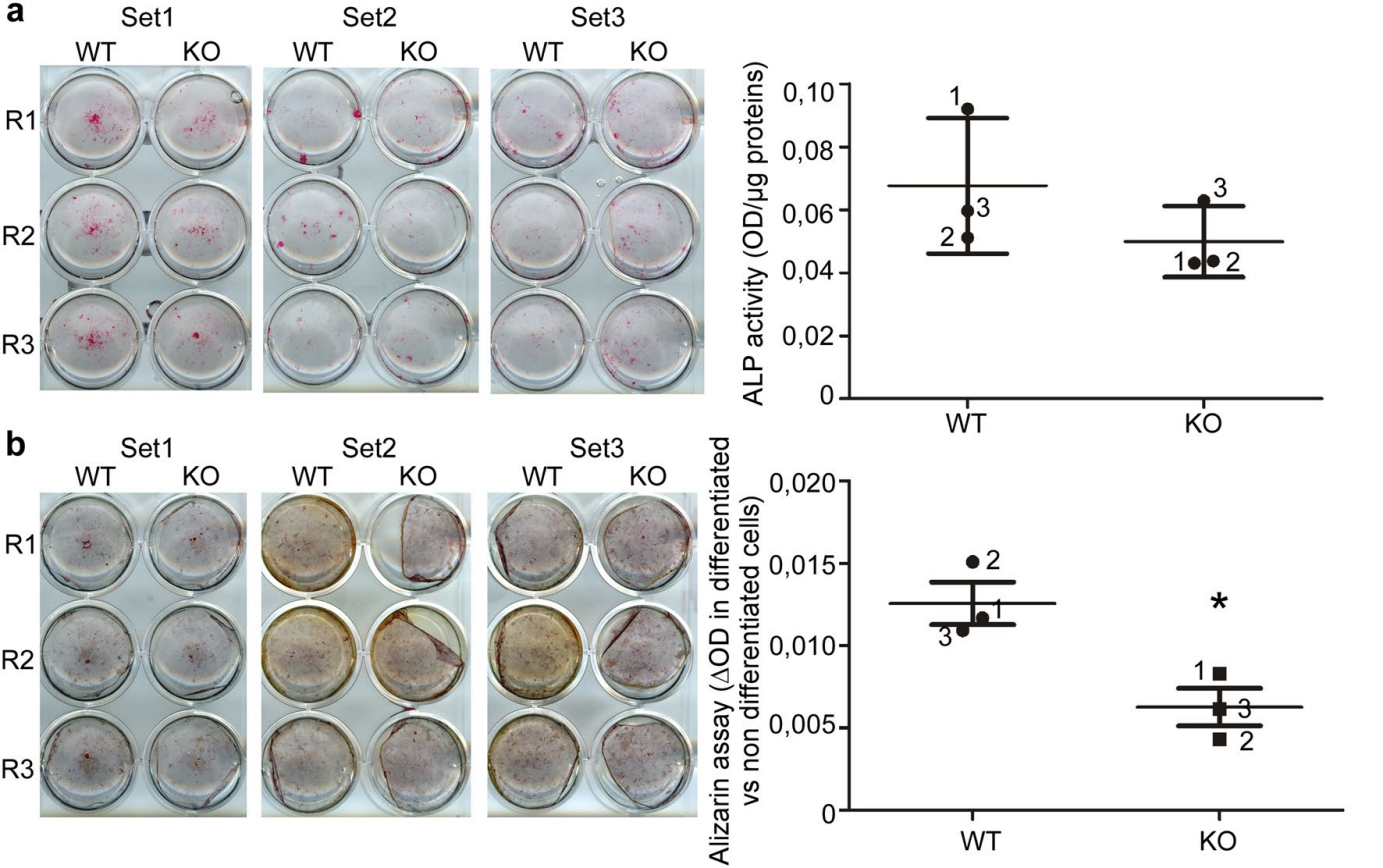
Analysis of the differentiation and activity level of *Hyal1* −/− osteoblasts cultured in vitro. Pre-osteoblasts were isolated from calvariae of new-born mice. Three independent sets, each derived from a pool of minimum 8 different calvariae, and comprising 3 replicates (R1 to R3), were analyzed. Differentiation was induced by supplementation of the culture medium with 50 µg/mL of vitamin C (Day 0 up to Day 14). From Day 15 to Day 21, the cells were cultured in the presence of 50 µg/mL vitamin C and 5 mM β-glycerophosphate. (**a**) Alkaline phosphatase staining of WT and *Hyal1* KO cells at Day 14, and measurement of alkaline phosphatase specific activity in cell lysates (Day 14). (**b**) Alizarin staining at Day 21 of differentiation, followed by lysis in NaOH and measurement of absorbance levels at 405 nm. The graphs show mean ± SEM values of the 3 independent sets. * p < 0.05. Paired t-test.

Lastly, we isolated osteoblast precursors from WT and *Hyal1* −/− newborn calvariae and differentiated them into osteoblasts in vitro for 21 days. To compare the differentiation rate of the cells, we detected alkaline phosphatase activity in the wells at Day 14 using a cytochemistry assay, and measured alkaline phosphatase specific activity after cell lysis (Fig. 8a). Three independent sets (each of them derived from a pool of minimum 8 different calvariae, and with 3 replicates per set), were analyzed. No statistically relevant differences between groups were detected for the alkaline phosphatase marker (p = 0.4). However, at day 21 of differentiation, an alizarin red staining followed by quantification of optical densities in tissue extracts at 405 nm (ΔOD relative to OD measured in extracts of non-differentiated controls) highlighted decreased mineralization by *Hyal1* −/− osteoblasts (Fig. 8b, p < 0.05).

## Discussion

Our results demonstrate that, in addition to osteoarthritis^12^, HYAL1 deficient mice exhibit altered bone homeostasis. The shorter femurs and thinner epiphyseal growth plates in aged *Hyal1* −/− mice suggest that HYAL1 plays a role in the longitudinal growth of long bones. As femur length is similarly decreased in mice deficient for another protein involved in HA depolymerization, CEMIP (previously known as KIAA1199)^17^, we can infer that undegraded HA accumulation in bone underlies these growth alterations. The major difference between the two models is that CEMIP is highly expressed in chondrocytes (much more than in osteoclasts) and CEMIP deficient mice display specific defects in endochondral ossification, whereas HYAL1 is highly overexpressed in osteoclasts^4^.

Since HA represents at most 7% of all bone glycosaminoglycans (GAGs), with GAGs making up less than 1% of all organic bone material^2,18^, the structural contribution of HA to BMD is probably very low. This might explain that HYAL1 deficiency does not result in increased BMD, by contrast to inactivation of cathepsin K, which causes pycnodysostosis^19^. On the other hand, our results highlight that endogenous HA controls BMD indirectly, through the regulation of bone cell behavior. *Hyal1* −/− mice exhibit decreased femur BMD compared to age-matched control mice. This phenotype appears accounted for, at least partly, by an increase of osteoclast numbers in the femur epiphysis and metaphysis and by an increase of osteoclast resorption activity. Little changes are detected in the diaphysis, which is not unexpected considering that cortical bone is less prone to remodeling than trabecular bone. CTX levels are elevated in *Hyal1* −/− sera compared to WT mice sera, as well as in the culture medium of *Hyal1*−/− osteoclasts differentiated on bone slices in vitro. Combined with the decreased PINP levels detected in *Hyal1* −/− plasma samples, decreased alkaline phosphatase staining in bone slices (close to the threshold of statistical relevance) and reduced mineralization by *Hyal1* −/− osteoblasts differentiated in vitro, these results demonstrate that HYAL1 deficiency potentiates bone resorption, while also decreasing bone formation by osteoblasts.

Other groups have reported that HA influences osteoclast and osteoblast function in vitro, in a size dependent manner (reviewed in^3^). Since the molecular mass pattern of HA molecules is not altered in other tissues of *Hyal1* −/− mice^8^, it seems more likely that the increased osteoclast numbers and activity, and decreased osteoblast activity, in the absence of HYAL1, results from a general accumulation of HA rather than from a change of its molecular mass in *Hyal1* −/− bones. Of note, it has been reported that HA binding to its cell surface receptor CD44 on bone marrow stromal cells increases the expression of RANKL, the cytokine that induces osteoclast differentiation^20^. HA accumulation around *Hyal1* −/− osteoclasts could also explain their increased resorption activity. Indeed, it has been shown that CD44 binding to extracellular HA activates podosomes formation and maturation into actin rings, a path that is known to promote bone degradation^21,22^. Consistent with these findings, *Cd44* knockout in mice results in smaller osteoclasts and shallow resorption pits^23,24^. Though actin ring numbers were found unchanged when *Hyal1* −/− osteoclasts were differentiated on bone slices, the increased resorption activity of these cells may result from a longer time spent attached to the bone due to CD44-HA pairing. Regarding osteoblasts, it has been reported that HA molecules of a broad range of molecular mass can increase their proliferation, differentiation, and activity in vitro^25^. However, our results suggest that endogenous HA accumulation in bones reduces bone matrix synthesis by osteoblasts. These findings are consistent with the report of Kaneko et al., who showed that HA negatively regulates osteoblastic differentiation by binding to CD44^26^.

Up until recently, only two lysosomal hydrolases, cathepsin K and TRAP, were found overexpressed upon osteoclast differentiation. Our past research revealed HYAL1 as a third lysosomal enzyme subject to specific upregulation during the differentiation process of osteoclasts from precursor cells^4^. The data presented here add another layer of understanding by showing that HYAL1 contributes to bone remodeling. HYAL1 affects both osteoclast and osteoblast function, most likely by controlling HA concentration in bones, and a lack of HYAL1 induces osteopenia. The metabolism of HA through hyaluronidases is also involved in chondrocytes differentiation^17^ and functions^27^. Hence, HA, despite its low abundance in bones, reveals itself as a fine regulator of bone structure and strength. In the future, the expression and activity of HYAL1, and the accumulation of HA, should be investigated in human bone pathological conditions, including osteoporosis and osteopetrosis, as they may represent novel pharmacological targets for medical use.

## Experimental procedures

### Animal experiments

*Hyal1* −/− mice (B6.129X1-*Hyal1*tm1Stn/Mmucd) obtained from MMRRC (Mutant Mouse Resource Research Centers, USA) were raised in our laboratory and backcrossed for 9 generations on a C57BL/6 genetic background. All experimental procedures were approved by the Animal Ethics Committee of the University of Namur and were performed in accordance with the relevant guidelines and regulations, as well as in compliance with the ARRIVE guidelines. The mice were housed at the UNamur animal facility and had free access to food and water. For one mouse group, an OVN food starvation period was included prior to blood collection for assays presented in Fig. 7. The protocols were recorded as JA14/214, JA20/356 and PM BO 21/015. Mice were anesthetized with 10 mg/kg xylazine and 100 mg/kg ketamine followed by euthanasia by cervical dislocation.

### pQCT

Femurs from 1-year-old C57BL/6 male mice were cleaned of soft tissues, fixed in 10% formalin for 48 h at room temperature (RT) and stored in 70% EtOH at 4 °C. The BMD was then determined by pQCT with a XCT Research Scanner. The bones were placed in a syringe filled with 70% EtOH and BMD was measured at three different sites identified by laying out from the most distal aspect of the femoral condyles a distance corresponding to 17.5, 22 and 50% of the total length of the femur. The slices had a thickness of 0.25 mm with a voxel size of 200 µm. The CALCBD program was used to analyze the trabecular and total BMD while the CORTBD function was used to assess the cortical BMD. In order to define trabecular bone, the outer 55% of the bone cross section was concentrically excluded by the CALCBD function. Tissues with a density below 200 mg/cm^3^ were not included in the analysis as they were considered as soft tissues by the software. The cortical bone parameters were determined with a threshold setting of 350 mg/cm^3^.

### Osteoclast differentiation in vitro

Bone marrow monocytes were isolated from 7-month-old C57BL/6 male mice and cultured for 5 days in Minimum Essential Medium Eagle alpha modified media (α-MEM, Lonza, Walkersville, MD, USA) containing 10% of inactivated FBS (Biological Industries, Beit-Haemek, Israel), 1/10th volume of L929 cell culture supernatant which contains M-CSF, 2 mM glutamine (Lonza, Walkersville, MD, USA), 100 U/mL penicillin and 100 µg/mL streptomycin (Lonza, Walkersville, MD, USA). The cells were then trypsinized and split in individual wells. The differentiation process was induced by incubation with 20 ng/mL of RANKL (R&D Systems, Minneapolis, MN, USA). The medium (which still contains M-CSF) was replaced every other day. Experiments were conducted on day 7, unless specified otherwise.

### Osteoblast differentiation in vitro

A minimum of 8 calvariae of 3–4 days old mice were used in individual preparations. Pre-osteoblasts were isolated by successive trypsin and collagenase treatments. Briefly, calvariae were first incubated for 20 min at 37 °C in Trypsin–EDTA (0.25%). Next, they were incubated for 20 min at 37 °C in DMEM/HamF12 (Gibco, Life Technologies, UK) without serum supplemented with 2% glutamine, 100 U/mL penicillin and 100 µg/mL streptomycin (Lonza, Walkersville, MD, USA), as well as 3 mg/mL collagenase D (Roche diagnostics Mannheim, Germany). After discarding of the supernatant, they were re-incubated twice for 45 min in a medium with the same composition. The cells isolated in these last two steps were then pooled, counted and cultured in DMEM/HamF12 (Gibco, Life Technologies, UK) supplemented with 2% glutamine, 100 U/mL penicillin and 100 µg/mL streptomycin, and 15% fetal bovine serum (80 000 cells/well in 12-well plates). 48 h later, the medium was replaced with DMEM/HamF12 (Gibco) supplemented with 2% glutamine, 100 U/mL penicillin and 100 µg/mL streptomycin, 2% UltraSer G (Satorius AG, Cergy Saint Christophe, France) and 50 µg/mL vitamin C (Merck, Darmstadt, Germany). From this day (Day 0), the medium was replaced every other day over a 21-day period. Of note, between Day 7 and Day 14, UltraSer G was replaced by 10% inactivated fetal bovine serum. From Day 14 to 21, the cells were cultured in the presence of 50 µg/mL vitamin C and 5 mM β-glycerophosphate (Merck KGaA, Darmstadt, Germany).

### TRAP staining of isolated osteoclasts

The cells were fixed for 30 s in 4.6 mM citric acid, 2.3 mM sodium citrate, 3 mM NaCl, 65% acetone and 3% formaldehyde. After washing with H_2_Od, the cells were incubated for 1 h at 37 °C in 0.07 mg/mL Fast Garnet GBC (Sigma-Aldrich, St. Louis, MO, USA) previously diazotized for 2.5 min with 100 mM sodium nitrite, 0.13 mg/mL Naphthol AS-BI Phosphoric Acid (Sigma-Aldrich, St. Louis, MO, USA), 10 mM tartrate and 100 mM acetate buffer at pH 5.2. The cells were then washed with H_2_Od and observed under a light microscope.

### Actin ring staining and bone resorption assay

Bone marrow monocytes cultured in the presence of M-CSF for 5 days were plated onto thin nasal bovine bone slices (kindly provided by Dr. Haibo Zhao, Center for Metabolic Bone Diseases, University of Arkansas for Medical Sciences, AR, USA). The differentiation into osteoclasts was then induced with RANKL as described above. 10 days later, the cells were fixed with 4% of paraformaldehyde in PBS, permeabilized with PBS-saponin 0.5% and incubated with 1% of bovine serum albumin in PBS. The cells were then incubated for 15 min at RT with 5% of Alexa 488-phalloidin in PBS (Invitrogen, Carlsbad, CA, USA) and, after several washes in PBS, for 20 min at RT with 1% of Hoechst 33258 in PBS (Molecular Probes, Eugene, OR, USA). These probes label actin and nuclei, respectively. Osteoclasts with a minimum of 3 nuclei were counted with a BX63 Olympus fluorescence microscope.

Soft brushing of the surface was subsequently conducted for 10 min in PBS containing 1% of Triton X-100 and followed by an incubation of 1 min in 1 M NaOH to complete the removal of the cells. Lastly, the bone slices were washed in H_2_Od, dehydrated in EtOH, dried and observed with a JEOL 6010LV scanning electron microscope to visualize resorption pits formed by osteoclasts.

### Alkaline phosphatase staining of osteoblasts differentiated in vitro

The Fast Red kit from Sigma was used, following the manufacturer’s recommendations.

### Alkaline phosphatase activity measured in osteoblasts differentiated in vitro

At day 14 of differentiation, the cells were lysed in PBS-Triton X-100 1% on ice. The lysates were then incubated for 15 min in the presence of 4 mM of p-nitrophenylphosphate (Sigma-Aldrich, St. Louis, MO, USA) in a 50 mM carbonate solution at pH 10 containing 5 mM of MgCl_2_. NaOH was then added (final concentration of 0.5 M), and the absorbance read at 405 nm with a Spectramax plate reader (Molecular Devices, CA, USA).

### Alizarin staining of osteoblasts differentiated in vitro

At day 21 of differentiation, the cells were fixed with PBS- PFA 4%, then incubated for 20 min in the presence of 1% Alizarin red solution (Sigma-Aldrich, St. Louis, MO, USA).

For signal quantification, after imaging of the wells using an Espon scanner, the tissues were dissolved in 10% acetic acid. The pH was adjusted between 4.1 and 4.3 with NH_4_OH and the absorbance was then read at 405 nm with a Spectramax plate reader (Molecular Devices, CA, USA).

### ELISA assays

The following kits were used, following manufacturer’s instructions: CrossLaps (CTX-I) ELISA kit, RatLaps (CTX-I) EIA kit, Rat/Mouse PINP EIA kit (Immunodiagnostic Systems, Tyne and Wear, UK).

### Histology—Immunohistochemistry

Femurs from C57BL/6 male mice were fixed with 4% paraformaldehyde in PBS for 24 h at 4 °C. The bones were successively washed with PBS, PBS-glycerol 5%, PBS-glycerol 10% and PBS-glycerol 15% (12 h incubation in each solution, at 4 °C). To decalcify bones, the samples were incubated for 12 days at 4 °C using 14.5% EDTA and 15% glycerol at pH 7.3. The specimens were then washed with PBS-glycerol 15%, PBS-glycerol 10%, PBS-glycerol 5% and PBS (12 h incubation in each solution, at 4 °C). After a step of dehydration in methanol and toluol, bones were embedded in paraffin and serially cut into 6 µm sections.

Sections were rehydrated and stained with HES. In a set of experiments, TRAP was detected as described above, followed by an hemalun staining on rehydrated bone tissue sections.

To detect HA, the sections were rehydrated, treated with 0.1 M glycine and with 3% H_2_O_2_ followed by a blocking step of 1 h in PBS-BSA 0.2% containing 0.02% of Triton X-100. The slices were then incubated for 1 h at RT with a biotinylated Hyaluronic Acid Binding Protein (HABP, 1:100 dilution, Merck Millipore, Burlington, MA, USA), washed with PBS-BSA 0.2%, and incubated for 1 h at RT with streptavidin-HRP (1:50 dilution, R&D Systems, Minneapolis, MN, USA). The 3,3ʹ-diaminobenzidine technique (Liquid DAB + Substrate chromogen System, Dako, Glostrup, Denmark) was used to reveal the signals.

To detect alkaline phosphatase (ALP), the slices were incubated for 1 h with a goat anti-ALP antibody (4 µg/mL final, AF2910 from R&D Systems, Minneapolis, MN, USA), followed by 1 h with a biotinylated secondary antibody (1:200), and for 1 h with streptavidin-HRP (1:50 dilution, R&D Systems, Minneapolis, MN, USA). All antibodies and streptavidin were prepared in PBS-BSA 0.2%. After washing, ALP signals were revealed using the 3,3’-diaminobenzidine technique (Liquid DAB + Substrate chromogen System, Dako, Glostrup, Denmark) and the slices were stained with hemalum.

### Quantifications and statistics

Quantifications were made using the ImageJ program (Rasband, W.S., ImageJ, U. S. National Institutes of Health, Bethesda, MD, USA, http://imagej.nih.gov/ij/). Statistical analyses of the data were performed with the GraphPad Prism 5.0 program (GraphPad Software, Inc., La Jolla, CA, USA).

## Abbreviations

ALP: Alkaline phosphatase
BMD: Bone mineral density
CTX: C-Terminal Telopeptide of type I-collagen
ECM: Extracellular matrix
HA: Hyaluronic acid
HABP: Hyaluronic acid binding protein
HES: Hematoxylin eosin saffron
KO: Knockout
M-CSF: Macrophage colony-stimulating factor
MM: Molecular mass
PINP: Procollagen type I N-terminal propeptide
pQCT: Peripheral quantitative computed tomography
RANKL: Receptor activator of nuclear factor kappa-B ligand
RT: Room temperature
SEM: Standard error of the mean
TRAP: Tartrate-resistant acid phosphatase
WT: Wild-type
